# Cord blood cell-derived iPSCs as a new candidate for chondrogenic differentiation and cartilage regeneration

**DOI:** 10.1186/s13287-017-0477-6

**Published:** 2017-01-28

**Authors:** Yoojun Nam, Yeri Alice Rim, Seung Min Jung, Ji Hyeon Ju

**Affiliations:** 1 CiSTEM Laboratory, Convergent Research Consortium for Immunologic Disease, Seoul St. Mary’s Hospital, College of Medicine, The Catholic University of Korea, Seoul, 137-701 Republic of Korea; 2 Division of Rheumatology, Department of Internal Medicine, Seoul St. Mary’s Hospital, Institute of Medical Science, College of Medicine, The Catholic University of Korea, #505, Banpo-Dong, Seocho-Gu, Seoul 137-701 Republic of Korea; 3 Division of Rheumatology, Department of Internal Medicine, College of Medicine, Yonsei University, Seoul, 120-749 Republic of Korea

**Keywords:** Induced pluripotent stem cells, Cord blood mononuclear cells, Chondrocytes, Cartilage regeneration, Regenerative medicine

## Abstract

**Background:**

The native articular cartilage lacks the ability to heal. Currently, ex vivo expanded chondrocytes or bone marrow-derived mesenchymal stem cells are used to regenerate the damaged cartilage. With unlimited self-renewal ability and multipotency, human induced pluripotent stem cells (hiPSCs) have been highlighted as a new replacement cell source for cartilage repair. Still, further research is needed on cartilage regeneration using cord blood mononuclear cell-derived hiPSCs (CBMC-hiPSCs).

**Methods:**

Human iPSCs were generated from CBMCs using the Sendai virus. The characterization of CBMC-hiPSCs was performed by various assays. Embryonic bodies (EBs) were obtained using CBMC-hiPSCs, and outgrowth cells were induced by plating the EBs onto a gelatin-coated plate. Expanded outgrowth cells were detached and dissociated for chondrogenic differentiation. Outgrowth cells were differentiated into chondrogenic lineage with pellet culture. Chondrogenic pellets were maintained for 30 days. The quality of chondrogenic pellets was evaluated using various staining and genetic analysis of cartilage-specific markers.

**Results:**

Reprogramming was successfully done using CBMCs. CBMC-hiPSCs (n = 3) showed high pluripotency and normal karyotype. Chondrogenic pellets were generated from the outgrowth cells derived from CBMC-hiPSC EBs. The generated chondrogenic pellets showed high expression of chondrogenic genetic markers such as ACAN, COMP, COL2A1, and SOX9. The production of extracellular matrix (ECM) proteins was confirmed by safranin O, alcian blue and toluidine blue staining. Expression of collagen type II and aggrecan was detected in the accumulated ECM by immunohistological staining. Chondrogenic pellets showed low expression of fibrotic and hypertrophic cartilage marker, collagen type I and X.

**Conclusions:**

This study reveals the potential of CBMC-hiPSCs as a promising candidate for cartilage regeneration.

**Electronic supplementary material:**

The online version of this article (doi:10.1186/s13287-017-0477-6) contains supplementary material, which is available to authorized users.

## Background

The articular cartilage is an elastic, white tissue that covers the end of bones and protects them from friction. Cartilage is mostly composed of chondrocytes and a large amount of extracellular matrix (ECM) rich in various types of collagen, proteoglycan and elastic fibers [1, 2]. Chondrocytes produce ECM components and trap themselves in a small room called ‘lacuna’, making it difficult to migrate and repair once the cartilage is damaged. Moreover, cartilage is an avascular tissue, i.e., it does not contain any blood vessels for nutrient supplementation. The avascularity of cartilage also hampers the migration of stem cells, reducing the regeneration potential of the tissue. These reasons indicate that it is almost impossible to naturally heal a damaged cartilage [3]. Therefore it is important to generate functional chondrocytes that can synthesize ECM in vitro or to obtain a fully developed cartilage for transplantation.

Cartilage reconstruction is usually done by transplantation using bone marrow-derived mesenchymal stem cells (BMSCs) or native chondrocytes isolated from the knee joint [4]. BMSCs and native chondrocytes have several advantages in regeneration capacity, because of their original chondrogenic potential. BMSCs are relatively easy to be obtained and already widely used as a cellular therapeutic material for various diseases including rheumatoid arthritis and osteoarthritis [5–9]. The slow rate of proliferation severely limits the regenerative potential of the cartilage [10–12]. It is challenging to obtain a high number of cells from in vitro culture of BMSCs or chondrocytes because extensive expansion can alter their phenotype [13–16]. Also, it was reported that the yield and the differentiation ability of BMSCs was reduced depending on the patient’s age and pathogenic conditions [17, 18]. In the case of native chondrocytes, additional damage to the knee joint is inevitable to obtain these cells. For these reasons, a new cell source for cartilage reconstruction is required.

It is important to generate hyaline cartilage for successful cartilage transplantation. Hyaline cartilage is the elastic type of cartilage, which is mostly made up of collagen type II. Long-term observation reveals that the transplanted chondrogenic cells tend to differentiate into hypertrophic chondrocytes that result in fibrotic cartilage-like phenotypes rather than hyaline cartilage [19, 20]. Although cell-based therapy is commonly practiced in clinic, it has not been proved that BMSCs and native chondrocytes can successfully repair the mature human cartilage [21, 22]. Other than cell-based therapy, mature cartilage transplantation by osteochondral grafting is another option to treat severely damaged cartilage. The advantage of osteochondral grafting is the transplantation of mature hyaline cartilage [23]. One part of the healthy cartilage of the joint is removed and transferred to the damaged region. Allogeneic cartilage transplantation has the risk of immunological rejection, which can eventually affect the viability of the allografts. Osteochondral autografting (mosaicplasty), however, is subject to the availability of healthy donor sites and the condition of the graft. These limitations still remain as an interesting topic in cartilage regeneration and require further studies by clinicians and researchers.

Human induced pluripotent stem cells (hiPSCs) have been highlighted as an alternative cell source for regenerative medicine. The discovery of hiPSCs provided a new strategy for drug screening and mechanistic studies of various diseases. Moreover, hiPSCs are a potential relevant cell source for the replacement of damaged tissues that have limited healing ability, such as the articular cartilage [24, 25]. Unlike BMSCs or native chondrocytes, the use of hiPSCs is highly recommended due to their high self-renewal ability and capacity to differentiate into targeted cells, including chondrocytes [26]. With the proper culture conditions, hiPSCs have greater potential as a replacement source to be used in cartilage regeneration.

In earlier reprogramming protocols, dermal fibroblasts were used for iPSC generation. Yet, the surgical biopsy procedure to obtain these cells restricted the opportunity for broad applications. Dermal fibroblasts are also reported to have higher genetic mutations due to exposure to environmental factors [27]. Blood cells were recommended as an alternative for reprogramming. Compared to fibroblasts, blood cells are a relatively accessible cell source. Blood cell-derived hiPSCs were successfully achieved with various reprogramming technologies [28–30]. These attempts were equally successful with cord blood mononuclear cells (CBMCs) [31–35].

In this study, we used CBMC-derived human iPSCs (CBMC-hiPSCs) for further experiments. Recently, CBMC-hiPSCs were successfully differentiated into cardiomyocytes and hepatocytes [17, 36, 37]. Previous work done by Guzzo et al., has shown the potential of CBMC-hiPSCs in chondrogenesis using micromass culture [38]. However, there is still much unknown about the chondrogenic regeneration ability of CBMC-hiPSCs, requiring further research. Chondrogenic pellets generated from CBMC-hiPSCs can be further used as a regenerative medicine in clinic. To achieve this goal, cartilage regeneration was attempted by pellet culture using CBMC-hiPSCs. Generated CBMC-hiPSCs were aggregated into embryoid bodies (EBs) and mesenchymal-like outgrowth cells were induced by attaching the EBs to a gelatin-coated dish. Chondrogenic pellets were generated using EB outgrowth cells.

Herein, we report the characteristics of cartilage-like chondrogenic pellets generated from CBMC-hiPSCs that indicate the possibility as a potential cell source for cartilage regeneration for future applications.

## Methods

### CBMC isolation

CBMCs were directly obtained from the Cord Blood Bank of the Seoul St. Mary’s Hospital. Cord blood was diluted with phosphate-buffered saline (PBS) and centrifuged through Ficoll gradient (GE Healthcare, Little Chalfont, UK) for 30 minutes at 850 × g. CBMCs were collected, washed, and frozen. Prior to being used, frozen CBMCs were thawed and resuspended in StemSpan medium (Stemcell Technologies, Vancouver, BC, Canada) supplemented with CC110 cytokine cocktail (Stemcell). Cells were maintained for 5 days at 5% CO_2_, 37 °C before reprogramming.

### Blood sample and ethics statement

The Institutional Review Board (IRB) of the Catholic University of Korea, Seoul St. Mary’s Hospital approved this study.

### Reprogramming using Sendai virus

The generation of hiPSCs was followed by the procedures mentioned in our previous work [35]. CBMCs were counted (3 × 10^5^) and seeded in one well of a 24-well plate. CytoTune-iPS Sendai Reprogramming Kit (Life Technologies, Carlsbad, CA, USA) was used to induce reprogramming. Transduction was performed with multiplicity of infection 7.5 per 3 × 10^5^ cell infectious units. After adding the viral components, cells were centrifuged at 1160 × g, 35 °C for 30 minutes and incubated at 5% CO_2_, 37 °C. On the following day, cells were transferred to a vitronectin-coated 12-well plate (Life Technologies) and settled by centrifugation for 10 minutes at 1160 × g, 35 °C. TeSR-E8 medium (Stemcell) was added (1:1) after centrifugation. Reprogrammed cells were maintained and expanded in TeSR-E8 media with daily media change.

### Alkaline phosphatase staining

To achieve colonies large enough for staining, 2 × 10^3^ cells were seeded in one well of a vitronectin-coated 6-well plate and expanded for 5–7 days. Staining of undifferentiated iPSC colonies was done using an alkaline phosphatase detection kit (EMD Millipore, Billerica, MA, USA). Cells were washed with PBS containing 0.05% Tween-20 (PBST; Biosesang, Seongnam, Korea) and fixed with 4% paraformaldehyde (Biosesang) for 2 minutes. Staining reagents including Fast Red Violet, Naphthol AS-BI phosphate solution, and water were mixed in a 2:1:1 ratio. Cells were washed twice with PBST. The staining solution mixture was treated for 15 minutes at room temperature (RT). After incubation, cells were washed with PBST and covered with PBS to prevent drying. Stained colonies were surveyed under the microscope.

### Immunocytochemical staining

To achieve large hiPSC colonies, 2 × 10^3^ cells were seeded per one well of a vitronectin-coated 6-well plate. Cells were expanded for 5–7 days with daily media change to induce large hiPSC colonies. After expansion, hiPSCs were washed with PBS and fixed with 4% paraformaldehyde. Cells were permeabilized using 0.1% triton X-100 (Biosesang) for 10 minutes. After permeabilization, cells were blocked for 30 minutes in RT with PBS containing 2% bovine serum albumin (BSA; Sigma-Aldrich, St. Louis, MO, USA) (PBA). Primary antibodies were diluted in PBA with the following dilution ratios; OCT4 (1/100; Santa Cruz Biotechnology, Dallas, TX, USA), KLF4 (1/250; Abcam, Cambridge, UK), SOX2 (1/100; BioLegend, San Diego, CA, USA), TRA-1-60 (1/100; EMD Millipore), TRA-1-81 (1/100; EMD Millipore), and SSEA4 (1/200; EMD Millipore). Primary antibodies were incubated for 2 hours at RT. Alexa Fluor 594- (1/400; Life Technologies) and 488-conjugated secondary antibodies (1/400; Life Technologies) were diluted in PBA and incubated for 1 hour at RT avoiding light. Cells were washed and mounted using ProLong Antifade mounting reagent (Thermo Fisher Scientific, Waltham, MA, USA). Stained colonies were detected with an immunofluorescence microscope.

### Polymerase chain reaction using CBMC-iPSC samples

5 × 10^5^ iPSCs were harvested and frozen in -20 °C. Total mRNA was extracted using Trizol (Life Technologies) and cDNA was synthesized using RevertAid™ First Strand cDNA Synthesis Kit (Thermo Fisher Scientific). Reverse transcriptase polymerase chain reaction was conducted using the synthesized cDNAs. Primer sequences are shown in the Table 1.

**Table 1:** Sequences of primers against pluripotent markers used in real time RT-PCR

Target name	Direction	Primer sequence	Size
OCT3/4	Forward	ACCCCTGGTGCCGTGAA	190
	Reverse	GGCTGAATACCTTCCCAAATA	
SOX2	Forward	CAGCGCATGGACAGTTAC	321
	Reverse	GGAGTGGGAGGAAGAGGT	
NANOG	Forward	AAAGGCAAACAACCCACT	270
	Reverse	GCTATTCTTCGGCCAGTT	
LIN28	Forward	GTTCGGCTTCCTGTCCAT	122
	Reverse	CTGCCTCACCCTCCTTCA	
DPPB5	Forward	CGGCTGCTGAAAGCCATTTT	215
	Reverse	AGTTTGAGCATCCCTCGCTC	
TDGF1	Forward	TCCTTCTACGGACGGAACTG	140
	Reverse	AGAAATGCCTGAGGAAAGCA	
GAPDH	Forward	GAATGGGCAGCCGTTAGGAA	414
	Reverse	GACTCCACGACGTACTCAGC	

### Karyotyping

Cells were cultured and expanded until 80% confluent. Chromosome resolution additive (Genial Genetic Solutions, Runcorn, UK) was added to each well. Cells were treated with Colcemid® for 30 minutes. Cells were harvested and treated with pre-warmed hypotonic solution. Fixation was performed with a 1:3 mixture of acetic acid to methanol solution. Slides were prepared for chromosome analysis using trypsin-Giemsa banding technique.

### Functional identification of iPSCs

To assess the three germ layer differentiation ability, a Human Pluripotent Stem Cell Functional Identification Kit was purchased (R&D Systems, Minneapolis, MN, USA). One day before the experiment, Cultrex PathClear BME (R&D Systems) was coated onto the culture dishes following the manufacturer’s instructions. Medium specific to each germ layer was prepared, and cells were cultured individually. After differentiation, cells were washed with PBS and fixed with 4% paraformaldehyde. Permeabilization and blocking was conducted with 0.3% Triton X-100 and 1% PBA for 45 minutes. Antibody against Otx2 (1/10; ectoderm), Brachyury (1/10; mesoderm), and Sox17 (1/10; endoderm) was diluted in PBA and incubated for 3 hours at RT. After washing the primary antibodies, Alexa Fluor 568 donkey anti-goat secondary antibody (1:200; R&D Systems) was diluted in PBA and incubated for 1 hour. Cells were washed with PBA, and treated with DAPI solution for 10 minutes in RT. Cells were washed and covered with PBS. Staining results were confirmed using fluorescence microscopy.

### EB-derived outgrowth cell induction

CBMC-hiPSCs were expanded and 2 × 10^6^ cells were prepared. Cells were resuspended in Aggrewell medium (Stemcell) and plated onto a 100-mm petri dish. Cells were incubated overnight at 5% CO_2_, 37 °C. The next day, media was changed into TeSR-E8 medium and cells were maintained for 6 days for expansion. After the expansion process, EBs were harvested and resuspended in DMEM containing 20% fetal bovine albumin (FBS) and placed on a gelatin-coated dish to induce outgrowth cells. Cells were maintained at 5% CO_2_, 37 °C for a week before chondrogenic differentiation.

### Chondrogenic differentiation using EB-derived outgrowth cells

Outgrowth cells derived from EBs were washed and detached from the dish. Cell clumps were removed by passing through a 40 μm cell strainer (Thermo Fisher Scientific). Single outgrowth cells were counted and 3 × 10^5^ cells per pellet were prepared. 3 × 10^5^ outgrowth cells were resuspended in chondrogenic differentiation medium (DMEM, 20% knockout serum replacement, 1 × non-essential amino acids, 1 mM L-glutamine, 1% sodium pyruvate, 1% ITS+ Premix, 10^-7^M dexamethasone, 50 mM ascorbic acid, 40 μg/mL L-proline supplemented with 50 ng/mL human bone morphogenetic protein 2 and 10 ng/mL human transforming growth factor beta 3) and transferred to a 15 mL conical tube. Cells were centrifuged at 750 × g for 5 minutes. Generated pellets were maintained for 30 days and media was changed every other day. BMSCs were used as a positive control.

### Histological analysis of chondrogenic pellet

Pellets were fixed in 4% paraformaldehyde for 2 hours at RT. One layer of gauze was placed onto a cassette and pellets were transferred onto the gauze. Dehydration was performed with sequential ethanol solutions. Dehydration solutions were cleared with graded ethanol and zylene mixtures (Duksan Pure Chemicals, Ansan, Korea) and paraffin was infiltrated overnight. The next day, pellets were fixed to a paraffin block and 7 μm sections were obtained using a microtome. Slides were dried for 10 minutes at 60 °C. Sections were deparaffinized by two cycles of zylene. Sections were rehydrated with decreasing sequential ethanol series and sections were rinsed under running tap water for 5 minutes. For alcian blue staining, sections were incubated in 1% alcian blue solution for 30 minutes. Slides were washed and counterstained with nuclear fast red for 1 minute. Safranin O staining was performed by incubating the slides in Weigert’s iron hematoxylin for 10 minutes. Slides were washed and incubated in 0.1% safranin O solution for 5 minutes. Finally, for toluidine staining, sections were incubated in 0.04% toluidine blue solution for 4 minutes. After the staining process, sections were washed and passed through increasing sequential ethanol series. Ethanol was cleared with two cycles of zylene and slides were mounted using VectaMount™ Permanent Mounting Medium (Vector Laboratories, Burlingame, CA, USA). Staining was confirmed under a bright field microscope.

### Immunohistochemistry

Sections were dried for 10 minutes at 60 °C and deparaffinized by two cycles of zylene. Sections were rehydrated with decreasing sequential ethanol series and sections were rinsed under running tap water for 5 minutes. Antigen unmasking was induced by incubating the sections in boiling citrate buffer (Sigma-Aldrich) for 15 minutes and leaving them to cool for 20 minutes. Cooled sections were then washed twice with deionized water (DW). The activity of endogenous peroxidase was blocked by incubating the sections in 3% hydrogen peroxide (Sigma-Aldrich) diluted in DW for 10 minutes. Sections were washed in DW twice, followed with additional washing with tris-buffered saline (TBS) containing 0.1% tween-20 (TBST). Sections were blocked for 20 minutes at RT with TBS containing 1% BSA. Primary antibodies diluted in blocking solution were added to the sections and incubated overnight at 4 °C. Primary antibodies were diluted in the following ratios: collagen type I (1/100; Abcam), collagen type II (1/100; Abcam) and Aggrecan (1/100; GeneTex, Irvine, CA, USA). Negative control slides were treated with the same amount of blocking solution without antibodies. The next day, sections were washed in TBST three times, each for 3 minutes, and secondary antibodies (1/200; Vector Laboratories) were applied for 40 minutes at RT. Sections were washed with TBST and incubated in ABC reagent (Vector Laboratories) for 30 minutes. Slides were washed with TBST three times and DAB solution (Vector Laboratories) was applied for 1 minute. Sections were washed in DW until the color was rinsed. Mayer’s hematoxylin (Sigma-Aldrich) was applied to the sections for 1 minute for counterstaining. Sections were washed and passed through increasing sequential ethanol series. Ethanol was cleared with two cycles of zylene and slides were mounted using VectaMount™ Permanent Mounting Medium (Vector Laboratories). Staining was confirmed under a bright field microscope.

### Polymerase chain reaction with chondrogenic pellet samples

Ten chondrogenic pellets were harvested on each time point and frozen at −80 °C. Samples were snap-frozen with liquid nitrogen and ground with a pestle. Ground pellet samples were incubated with Trizol for mRNA extraction. From the extracted mRNAs, cDNA was synthesized and polymerase chain reaction was conducted with primers against chondrocyte-specific markers. Primer sequences for RT-PCR are provided in Table 2. Primer sequences for real time-PCR are provided in Table 3. Mean cycle threshold values from triplicate experiments were used to calculate the gene expression normalized to GAPDH as an internal control.

**Table 2:** Sequences of primers against chondrogenic markers in RT-PCR

Target name	Direction	Primer sequence	Size
SOX9	Forward	GAACGCACATCAAGACGGAG	631
	Reverse	TCTCGTTGATTTCGCTGCTC	
ACAN	Forward	TGAGGAGGGCTGGAACAAGTACC	349
	Reverse	GAGGTGGTAATTGCAGGGAACA	
COL2A1	Forward	TTCAGCTATGGAGATGACAATC	472
	Reverse	AGAGTCCTAGAGTGACTGAG	
COMP	Forward	CAACTGTCCCCAGAAGAGCAA	588
	Reverse	TGGTAGCCAAAGATGAAGCCC	
COL1A1	Forward	CCCCTGGAAAGAATGGAGATG	148
	Reverse	TCCAAACCACTGAAACCTCTG	
COL10	Forward	CAGTCATGCCTGAGGGTTTT	196
	Reverse	GGGTCATAATGCTGTTGCCT	
GAPDH	Forward	GAATGGGCAGCCGTTAGGAA	414
	Reverse	GACTCCACGACGTACTCAGC

**Table 3:** Sequences of primers against chondrogenic markers in real time-PCR

Target name	Direction	Primer sequence	Size
SOX9	Forward	TTCCGCGACGTGGACAT	77
	Reverse	TCAAACTCGTTGACATCGAAGGT	
ACAN	Forward	AGCCTGCGCTCCAATGACT	107
	Reverse	TAATGGAACACGATGCCTTTCA	
COL2A1	Forward	GGCAATAGCAGGTTCACGTACA	79
	Reverse	CGATAACAGTCTTGCCCCACTTA	
COMP	Forward	AGCAGATGGAGCAAACGTATTG	76
	Reverse	ACAGCCTTGAGTTGGATGCC	
COL1A1	Forward	CCCCTGGAAAGAATGGAGATG	148
	Reverse	TCCAAACCACTGAAACCTCTG	
COL10	Forward	CAGTCATGCCTGAGGGTTTT	196
	Reverse	GGGTCATAATGCTGTTGCCT	

## Results

### Generation of hiPSCs using isolated CBMC

**Fig. 1:**
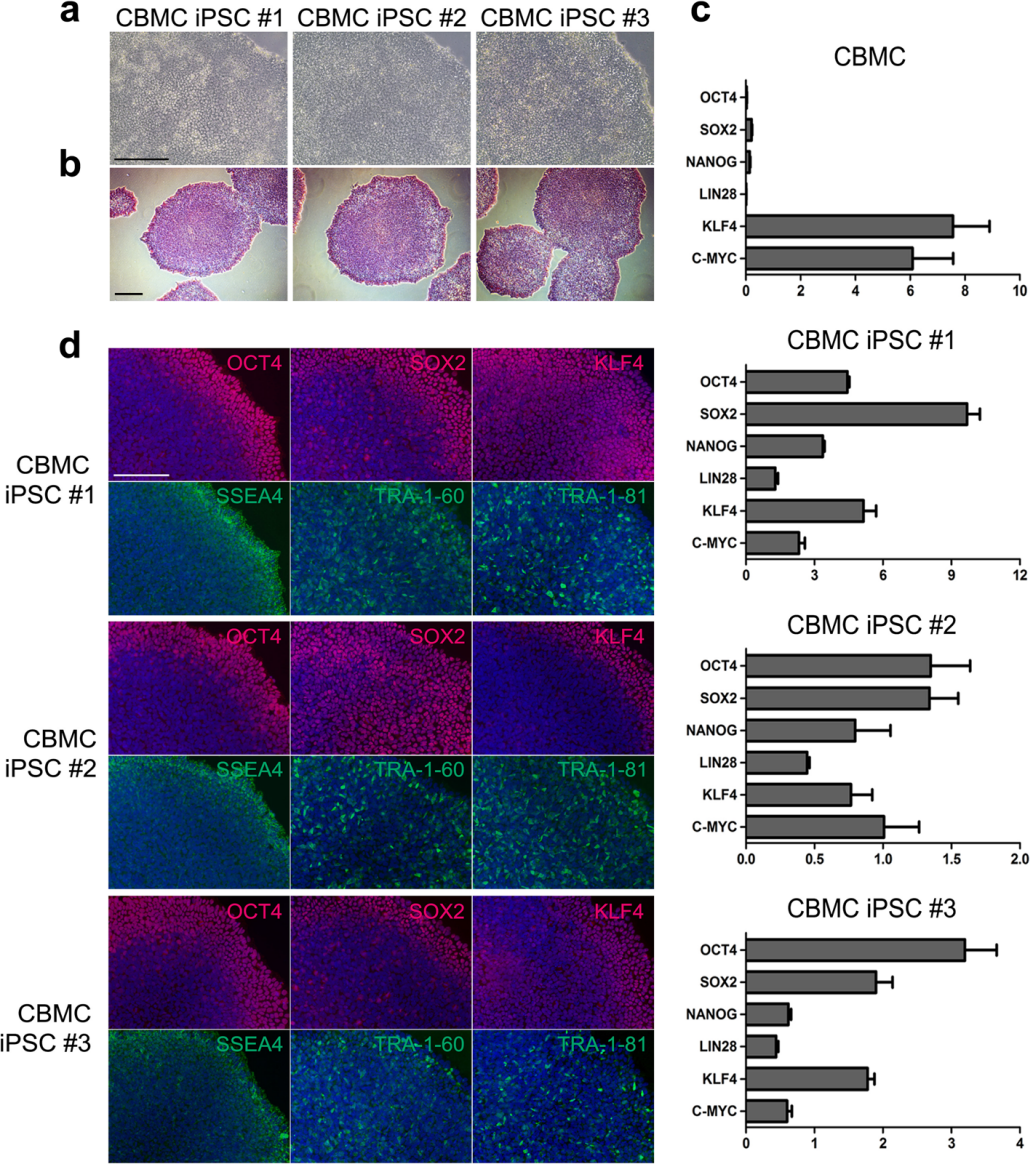
Characterization of three CBMC-hiPSC lines. **a** Morphology of the three generated CBMC-hiPSC lines. **b** CBMC-hiPSC colonies positively stained with alkaline phosphatase. **c** Relative expression of pluripotent markers in each CBMC-hiPSC line. Primary CBMC was used as a control. **d** Immunofluorescence staining image of the generated CBMC-hiPSC lines. All scale bars represent 200 μm. *CBMC-hiPSC* cord blood mononuclear cell-derived human induced pluripotent stem cell

Reprogramming of CBMC was induced using Sendai virus containing Yamanaka factors. With several passages after transduction, CBMC-hiPSCs showed embryonic stem cell-like colonies during expansion (Fig. 1a). CBMC-hiPSCs were maintained and purified until cell lines had homogenous cell morphology. Homogenous CBMC-hiPSCs were used for further characterization. Established CBMC-hiPSCs exhibited positive alkaline phosphatase staining (Fig. 1b). The relative expression of pluripotent markers including OCT4, SOX2, NANOG, LIN28, KLF4, and c-MYC was assessed (Fig. 1c). Parental CBMC was used as a negative control. The expression of OCT4, SOX2, NANOG, and Lin28 was increased in CBMC-hiPSCs. The expression of KLF4 and c-MYC, however, was lower in CBMC-hiPSCs than that of CBMCs. Typical cell surface markers (SSEA4, OCT4, SOX2, KLF4, TRA-1-80, and TRA-1-60) were confirmed by immunocytochemical analysis (Fig. 1d). All generated cell lines expressed the canonical markers of pluripotency. We confirmed that CBMC-hiPSCs maintained normal karyotypes after the reprogramming process (see Additional file 1a). CBMC-hiPSCs were also able to differentiate into each germ layer (see Additional file 1b). Together, these data suggested that CBMC-hiPSCs were successfully generated and the cells exhibited the genuine characteristic of pluripotency.

### Chondrogenic differentiation of CBMC-iPSCs

**Fig. 2:**
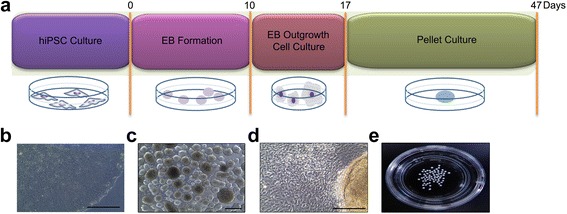
Chondrogenic pellet generation using CBMC-hiPSCs. **a** Scheme of chondrogenic pellet generation. **b** Morphology of CBMC-hiPSC. **c** Morphology of generated EBs. **d** Image of outgrowth cells derived from EBs attached to a gelatin-coated culture dish. **e** Image of chondrogenic pellets. All scale bars represent 200 μm. *EB* embryoid body, *hiPSC* human induced pluripotent stem cell

To confirm the cartilage regeneration ability of CBMC-hiPSCs, we performed chondrogenic differentiation through EB culture and outgrowth cell induction. A simple scheme of the chondrogenic pellet generation process is shown in Fig. 2a. Colonies of CBMC-hiPSCs were prepared for chondrogenic differentiation (Fig. 2b). CBMC-hiPSCs were expanded and aggregated into EBs (Fig. 2c). EBs were enlarged for several days and transferred to gelatin-coated dishes to induce outgrowth cells (Fig. 2d). Outgrowth cells were expanded and dissociated into single cells for chondrogenic differentiation. Using 2 × 10^6^ iPSCs, numerous chondrogenic pellets were obtained (Fig. 2e). After 30 days of differentiation, chondrogenic pellets were generated using EB outgrowth cells. The generated chondrogenic pellets exhibited a three-dimensional spheroid configuration. Throughout this process, we confirmed that CBMC-hiPSCs were able to differentiate into chondrocytes and formed a spheroid-shaped cartilage-like appearance by ECM accumulation.

### Confirmation of chondrogenic gene expression

**Fig. 3:**
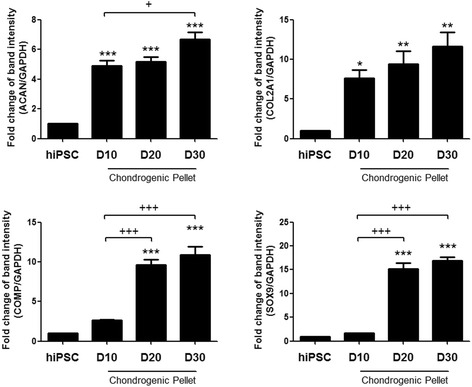
Genetic characterization of chondrogenic pellet generated from CBMC-hiPSCs. The expression of COL2A1, ACAN, COMP, and SOX9 in day 10, 20, and 30 chondrogenic pellets. Data was obtained using RT-PCR and band intensity was evaluated. (^*, +^ *p* < 0.05, ^**^, ^++^ *p* < 0.01, ^***, +++^ *p* < 0.001). *ACAN* aggrecan gene, *COL2A1* collagen type II gene, *COMP* cartilage oligomeric matrix protein gene, *hiPSC* human induced pluripotent stem cell, *SOX9* sex-determining region Y-box 9 gene

Previously, chondrogenic pellets were successfully generated from CBMC-hiPSCs. Also, the differentiated cells were able to synthesize ECM components and exhibit cartilage-like features. We examined the gene expression of major ECM component proteins such as aggrecan (ACAN), collagen type II (COL2A1), and cartilage oligomeric matrix protein (COMP) on several time points (day 10, 20, and 30). The increasing expression of ACAN, COL2A1, and COMP was confirmed (Fig. 3). Sex-determining region Y-box 9 (SOX9) is known as an early chondrogenic marker and a transcription factor that regulates the expression of ECM protein genes. The expression of SOX9 significantly increased after day 20. According to these results, we confirmed the genetic characteristics of the generated chondrogenic pellets. Corresponding to the cartilage-like morphology, the increased gene expression of major ECM component proteins was confirmed.

### Histological characterization of chondrogenic pellets

**Fig. 4:**
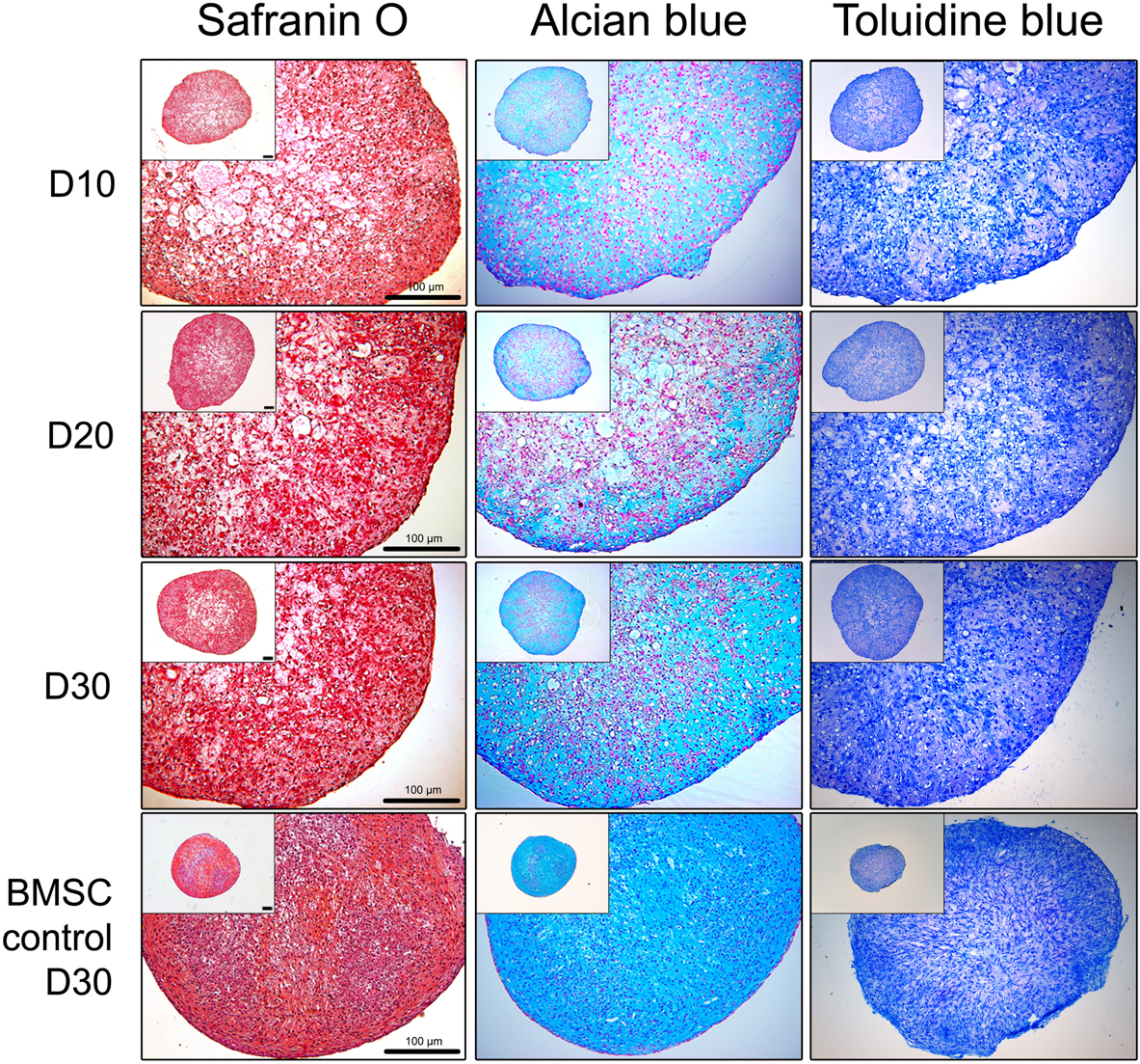
Histological analysis of CBMC-hiPSC-derived chondrogenic pellet. Image of pellets stained by safranin O, alcian blue, and toluidine blue on day 10, 20, and 30. All scale bars represent 100 μm. *BMSC* bone marrow-derived mesenchymal stem cell

According to the confirmation of increased chondrogenic marker expression, the protein levels of the chondrogenic pellets generated from CBMC-hiPSCs were evaluated by histological analysis (Fig. 4). Safranin O, alcian blue, and toluidine blue staining are authorized staining methods for ECM detection in cartilage. With the staining results, we confirmed ECM accumulation at the lining of the pellets even on the early stage of differentiation (day 10). Lacunae are one of the major features shown in the articular cartilage. Hollow lacuna-like capacities were visible after day 10. The size, however, decreased as differentiation progressed. On day 30 of differentiation, as the ECM was accumulated in the capacities, it appeared more like a lacuna in the articular cartilage. The staining intensity of day 30 pellets was almost similar to that of MSC controls.

**Fig. 5:**
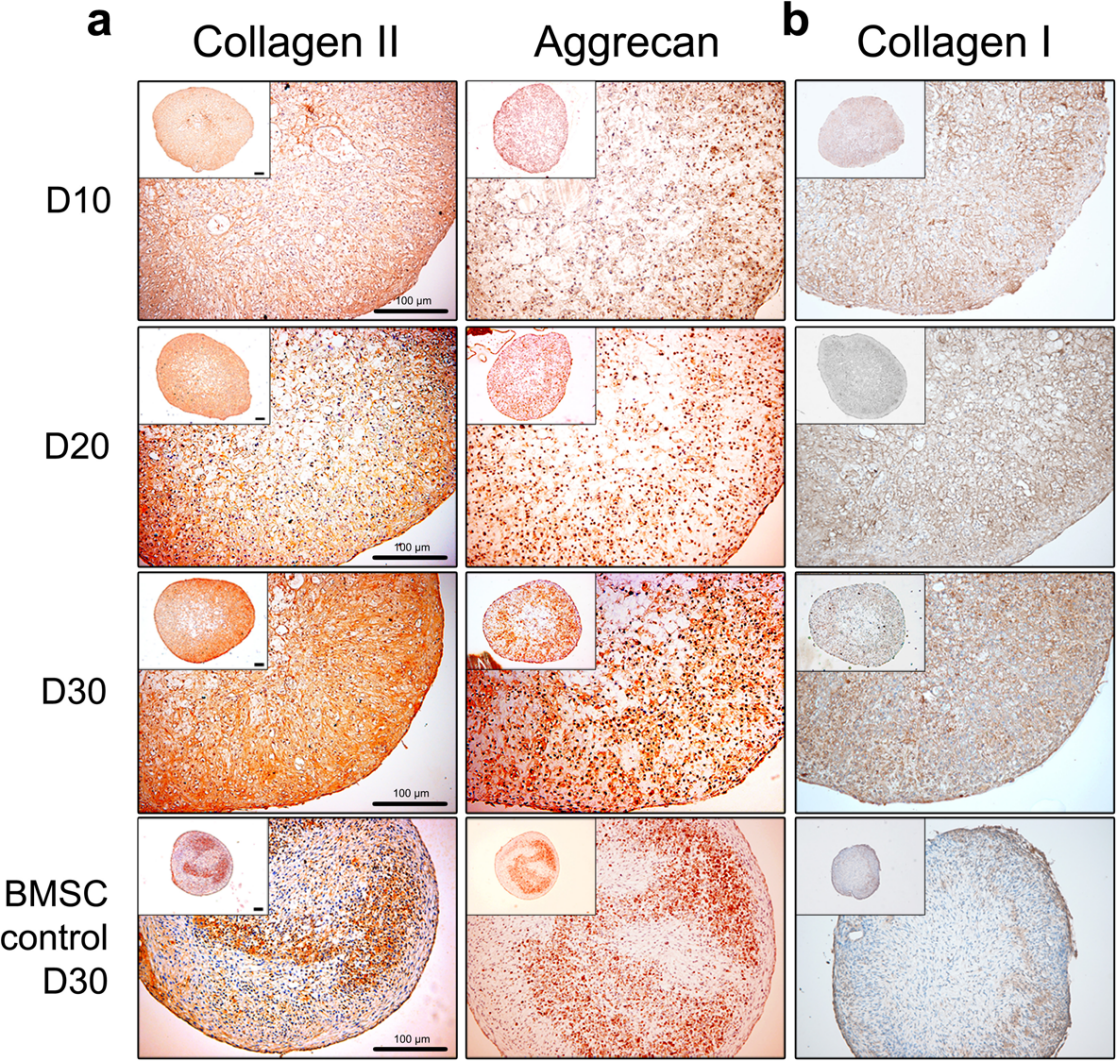
Immunohistological analysis of CBMC-hiPSC-derived chondrogenic pellets. **a** Image of pellet harvested at various time points stained with antibody against collagen type II and aggrecan. **b** Image of pellet stained with antibody against collagen type I. All scale bars represent 100 μm. *BMSC* bone marrow-derived mesenchymal stem cell

The quality of cartilage is dependent on the major type of ECM proteins. Therefore, it is important to identify the specific proteins that comprise the ECM. Aggrecan and collagen type II proteins are known as the major components that constitute the ECM. Collagen type II is the dominant collagen type that represents the hyaline cartilage. We specifically stained chondrogenic pellets with antibody against collagen type II and aggrecan (Fig. 5a). The staining intensity of collagen type II was higher in CBMC-hiPSC-derived chondrogenic pellets than that of MSC controls. Corresponding to the previous staining results, aggrecan and collagen type II was mostly detected at the pellet lining on day 30. The major characteristic of fibrotic cartilage is the high expression of collagen type I. We confirmed that the generated pellets did not have dominant characteristics of the fibrotic cartilage (Fig. 5b). The expression of collagen type I was relatively higher than that of MSC control pellets. Yet, the expression maintained a constant level and did not significantly increase during differentiation. Taken all together, chondrogenic pellets generated from CBMC-hiPSCs featured similar qualities of pellets derived from MSCs after 30 days of differentiation. Chondrocytes differentiated from CBMC-hiPSCs were able to produce ECM component proteins. CBMC-hiPSC-derived chondrogenic pellets had higher expression of collagen type II than that of collagen type I. In conclusion, we confirmed that CBMC-hiPSCs were able to generate cartilage-like features, which were similar to the characteristics of hyaline cartilage.

### Further analysis of genetic markers in chondrogenic pellets derived from CBMC-hiPSCs and MSCs

**Fig. 6:**
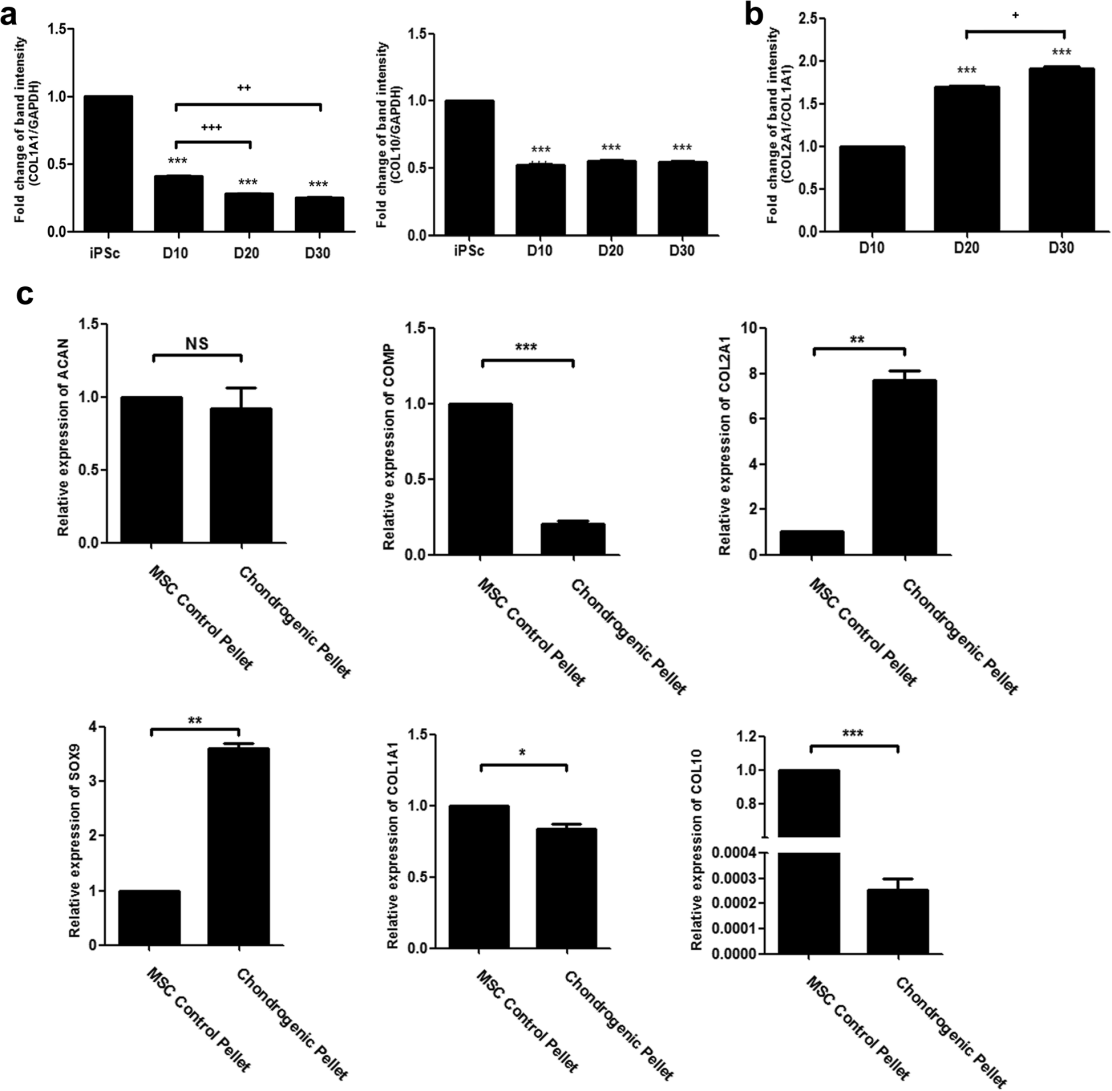
Further analysis of genetic markers in chondrogenic pellets derived from CBMC-hiPSCs and MSCs. **a** The expression of fibrotic cartilage representative gene, COL1A1 and hypertrophic marker, COL10 at various time points. **b** The ratio of COL2A1 and COL1A1 at day 10, 20, and 30. **c** The relative expression of ACAN, COMP, COL2A1, SOX9, COL1A1, and COL10 in chondrogenic pellets derived from BMSC and CBMC-hiPSC on day 30. (^*,+^ *p* < 0.05, ^**,++^ *p* < 0.01^, ***,+++^ *p* < 0.001). *ACAN* aggrecan gene, *COL10* collagen type 10 gene, *COL1A1* collagen type I gene, *COL2A1* collagen type II gene, *COMP* cartilage oligomeric matrix protein gene, *iPSC* induced pluripotent stem cell, *MSC* mesenchymal stem cell, *SOX9* sex-determining region Y-box 9 gene

Collagens are the most abundant proteins that compose the ECM. Various types of collagen exist, however, collagen type I, II, and X are mainly related to the cartilage. Previously, we confirmed the expression of collagen type I and type II by histochemical analysis (Fig. 5a and b). Based on these results, the expression of collagen type I gene (COL1A1) was further analyzed (Fig. 6a). The gene expression of collagen type X (COL10), a protein known as the dominant type expressed in hypertrophic cartilage, was analyzed as well. We confirmed the steady expression of collagen type I with histochemical staining. The expression of COL1A1, however, decreased at each time point. The expression of COL10 was not altered during differentiation. As mentioned earlier, the ratio of collagen type I to II can alter the outcome characteristic of the chondrogenic pellet. Using the previous gene expression data, we evaluated the gene expression ratio of COL2A1 to COL1A1 (Fig. 6b). The overall increasing ratio indicates higher expression of hyaline cartilage gene against fibrotic cartilage gene.

CBMC-hiPSC-derived chondrogenic pellets was compared with chondrogenic pellets generated from BMSCs on day 30 using real time-PCR (Fig. 6c). There was no statistical significance of ACAN expression between the two samples. The expression of COL2A1 and SOX9 was significantly higher in the chondrogenic pellets differentiated from CBMC-hiPSCs compared to BMSC-derived pellets. Yet, COMP was highly expressed in MSC control pellets. The expression of the fibrotic marker, COL1A1, was higher in MSC control pellets as well. The expression of the hypertrophic marker COL10, however, was remarkably lower in the CBMC-hiPSC-derived chondrogenic pellets. These results highlight the possibility of CBMC-hiPSCs as a potential cell source for cartilage regeneration and further future applications.

## Discussion

The repair of articular cartilage injury caused by joint-related diseases, external wound, or trauma still remains an unsolved clinical issue. Human iPSCs opened new possibilities for regeneration of personalized medicine. Numerous groups attempted chondrogenesis using hiPSCs generated from various somatic cell types [38–42].

We reprogrammed CBMCs into hiPSCs using Sendai viral vectors containing Yamanaka factors (Fig. 1a) [35]. Due to the established cord blood banking system around the world, CBMCs are widely utilized. The conversion of cord blood banks into hiPSC banks for allogeneic regenerative medical treatment holds tremendous potential and possibilities [43]. With the database of HLA phenotypes, a CBMC-based hiPSC banking system can give a new efficient strategy for cell-based therapy by generating cell lines with homozygous HLA-typed hiPSCs. Homozygous cell lines can be widely utilized in treatment with minimum material or cell lines. This concept can be equally applied to cartilage regeneration. Generation of cartilage using HLA-homozygous hiPSC lines can eliminate the immunological reactions when applied in allograft transplantation. Therefore, the use of CBMC-hiPSCs can be highly efficient for future application in cartilage transplantation. Many reports demonstrated that the origin of the somatic cell used in reprogramming, can affect the developmental and differential ability [44–46]. Guzzo et al. highlighted the potential of CBMC-hiPSCs for chondrogenic differentiation [38]. This work was done with one CBMC-hiPSC line and two articular chondrocyte-derived hiPSC lines. However, additional study is required to further identify CBMC-hiPSCs as an ideal cell source for cartilage regeneration and transplantation. We attempted chondrogenic differentiation using CBMC-hiPSCs (n = 3) and analyzed the cartilage regeneration ability by various assays. For future application in transplantation, chondrogenic differentiation was performed by pellet culture.

Prior to the differentiation process, the characteristics of the generated CBMC-hiPSCs were analyzed. The undifferentiated stem cell characteristic was confirmed by the positive staining against alkaline phosphatase (Fig. 1b). Increased expression of pluripotent markers was confirmed by immunocytochemical and cytogenetic assays (Fig. 1c and d). All cell lines were able to differentiate into each germ layer and featured a normal karyotype (see Additional file 1).

Chondrogenic differentiation is performed through two critical steps: induction of mesenchymal-like outgrowth cells and chondrogenic pellet formation. Previous studies confirmed that outgrowth cells generated from hiPSCs are functionally and molecularly similar to native MSCs [47, 48]. These studies used monolayer culture or EB culture as a pre-differentiation step to induce mesenchymal-like progenitor cells [48, 49]. Direct differentiation using monolayer culture, however, was time-consuming compared to the use of EBs. The morphology and differentiation potential of mesenchymal-like progenitor cells also differed greatly according to the cell density [50].

Based on these considerations, we used EBs to induce mesenchymal-like outgrowth cells (Fig. 2a). Using only 2 × 10^6^ cells, a large amount of EBs was prepared (Fig. 2b). To generate outgrowth cells, maintained EBs were plated onto gelatin-coated dishes for expansion. Seeding 50–75 EB per cm^2^ was adequate for the proper density of outgrowth cells (Fig. 2c). Chondrogenic pellets were generated by pellet culture using the outgrowth cells (Fig. 2d). We were able to obtain 50–100 pellets in total (Fig. 2e). The quality of the generated chondrogenic pellets, however, is as important as the quantity. The expression of several specific genes for ECM proteins was confirmed. ACAN is a proteoglycan that forms aggregates in the ECM, and induces the interaction with hyaluronan [51]. COL2A1 is the fundamental protein for hyaline cartilage that represents the non-hypertrophic characteristic of a healthy cartilage [52]. The expression of ACAN and COL2A1 significantly increased on day 10 (Fig. 3). The expression of COMP, which is a non-collagenous ECM protein started to increase sharply on day 20. It was reported that the overexpression of COMP increased the levels of ACAN and COL2A1, however, the expression of the early chondrogenic marker, SOX9, was not affected by the increased COMP expression [53]. In our study, the expression of SOX9 increased in the same pattern as COMP. By several cartilage-specific staining methods, we confirmed that the ECM components were accumulated as much as those of cartilage generated from MSCs on day 30 (Fig. 4).

After generating cartilage, it is critical to distinguish hyaline cartilage from fibrotic cartilage. Fibrotic, or hypertrophic cartilage, is a further mature type that tends to differentiate into bone [54]. The generated chondrogenic pellet from CBMC-hiPSCs showed relatively low expression of hypertrophic markers (Fig. 6a). Expression of COL1A1 was decreasing while COL2A1 expression increased throughout differentiation (Fig. 6b).

The property of chondrogenic pellets derived from CBMC-hiPSCs was compared to that of pellets derived from BMSCs on day 30 (Fig. 6c). The expression of COL2A1 and SOX9 significantly increased in pellets generated from CBMC-hiPSCs. Interestingly, the expression of COMP was higher in the chondrogenic pellets generated from BMSCs. Also, the expression of COL1A1 and COL10 was increased in pellets derived from BMSCs as well. COMP is thought as one of the essential proteins that exist in the tendon and cartilage. It provides adaptor function and binds directly to collagen type I and XII, which is a protein that decorates the surface of collagen I fibrils [55]. It is also considered as a marker of cartilage breakdown [56]. Recently, COMP is also thought as a biological marker for several diseases with high cartilage turnover rates such as rheumatoid arthritis and systemic sclerosis [54, 56–58]. This indicates that CBMC-hiPSCs might have less fibrotic or hypertrophic cartilage properties compared to BMSC-derived chondrogenic pellets.

We confirmed that the cartilage regenerated using CBMC-hiPSCs showed a healthy phenotype and can be considered a material for tissue regeneration. Yet, an improved method with a shorter differentiation time line is required for further applications. Further development of quality control standards to validate cartilage with higher hyaline is also required for future applications of CBMC-hiPSCs as cell material for cartilage regeneration.

## Conclusions

CBMC-hiPSCs can be used as a material for cartilage regeneration. CBMC-hiPSCs can open new possibilities for personalized regenerative medicine.

## Additional file

Additional file 1: Further characterization of three hiPSC lines generated from CBMCs. **a** Karyotype images of the generated CBMC-hiPSCs. **b** Immunofluorescence image of CBMC-hiPSCs differentiated to ectoderm (Otx2), mesoderm (Brachyury) and endoderm (Sox17). All scale bars represent 200 μm. (PDF 278 kb)

## Abbreviations

ACAN: Aggrecan gene
BMSC: Bone marrow-derived mesenchymal stem cell
CBMC: Cord blood mononuclear cell
CBMC-hiPSC: CBMC-derived human iPSC
COL10: Collagen type 10 gene
COL1A1: Collagen type I gene
COL2A1: Collagen type II gene
COMP: Cartilage oligomeric matrix protein gene
EB: Embryoid body
ECM: Extracellular matrix
hiPSC: Human induced pluripotent stem cell
SOX9: Sex-determining region Y-box 9 gene
