# Supplementary material for: Cord blood cell-derived iPSCs as a new candidate for chondrogenic differentiation and cartilage regeneration
Source: Stem Cell Res Ther. 2017 Jan 28;8:16. doi: 10.1186/s13287-017-0477-6 (PMC5273802; doi:10.1186/s13287-017-0477-6)
Supplement: Additional file 1: — Further characterization of three hiPSC lines generated from CBMCs. a Karyotype images of the generated CBMC-hiPSCs. b Immunofluorescence image of CBMC-hiPSCs differentiated to ectoderm (Otx2), mesoderm (Brachyury) and endoderm (Sox17). All scale bars represent 200 μm. (PDF 278 kb) [file 13287_2017_477_MOESM1_ESM.pdf]

Additional file 1

Cord Blood Cell-derived iPSCs as a New Candidate for Chondrogenic Differentiation and Cartilage Regeneration

Yoojun Nam[1,2], Yeri Alice Rim[1,2], Seung Min Jung[3] and Ji Hyeon Ju[1,2,*]

Contact Information

[1] *CiSTEM laboratory, Convergent Research Consortium for Immunologic Disease, Seoul St. Mary's Hospital, College of Medicine, The Catholic University of Korea, Seoul, 137-701, Republic of Korea;*

[2] *Division of Rheumatology, Department of Internal Medicine, Seoul St. Mary's Hospital, Institute of Medical Science, College of Medicine, The Catholic University of Korea, Seoul, 137-701, Republic of Korea;*

[3] *Division of Rheumatology, Department of Internal Medicine, College of Medicine, Yonsei University, Seoul, 120-749, Republic of Korea;*

Yoojun Nam: givingtreemax@gmail.com

Yeri Alice Rim: llyerill0114@gmail.com

Seung Min Jung: jsmin00@yuhs.ac

*To whom correspondence should be addressed:

Ji Hyeon Ju, MD, PhD

Division of Rheumatology, Department of Internal Medicine, Seoul St. Mary's Hospital, College of Medicine, The Catholic University of Korea, #505, Banpo-Dong, Seocho-Gu, Seoul, Republic of Korea. 137-701
Telephone: +82 2 2258 6893; Fax: +82 2 3476 2274;
E-mail address: juji@catholic.ac.kr

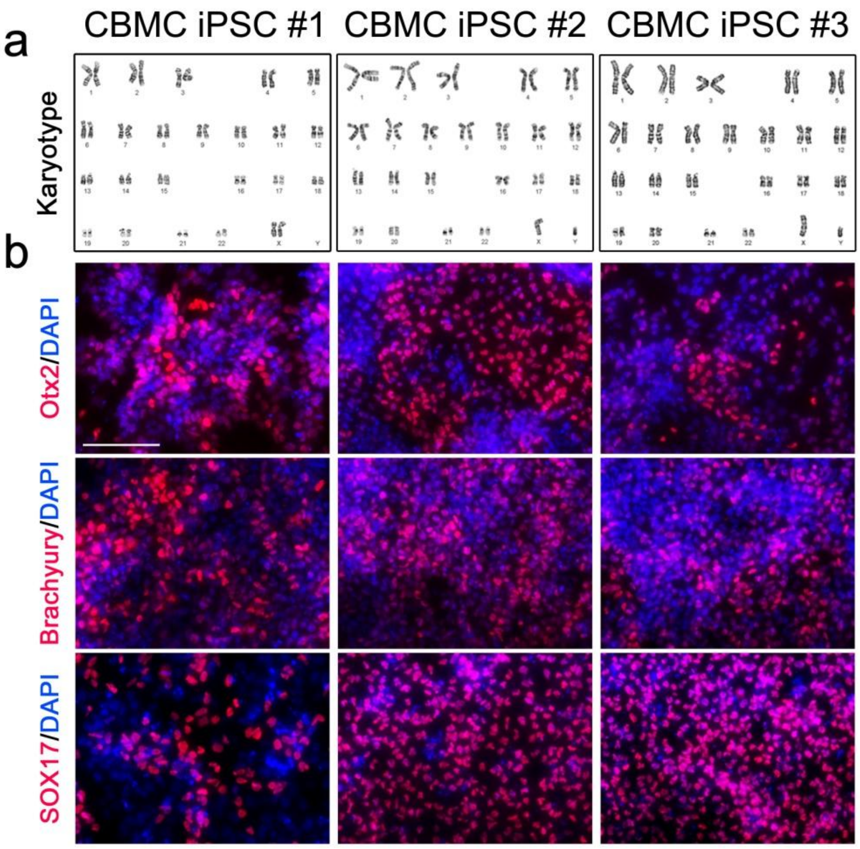

Additional file 1:
Further characterization of three hiPSC lines generated from CBMCs.
a Karyotype images of the generated CBMC-hiPSCs. **b** Immunofluorescence image of CBMC-hiPSCs differentiated to ectoderm (Otx2), mesoderm (Brachyury) and endoderm (Sox17). All scale bars represent 200 µm.
